# From blood to brain: postmortem brain concentrations and brain–blood ratios of chlorprothixene, clozapine, olanzapine, and quetiapine

**DOI:** 10.1093/jat/bkag065

**Published:** 2026-07-16

**Authors:** Mikayla Z van der Meer, Brian S Rasmussen, Michael Nedahl, Marie K K Nielsen

**Affiliations:** Faculty of Health Sciences, Forensic Medicine, University of Copenhagen, Copenhagen, DK 2100, Denmark; Faculty of Health Sciences, Forensic Medicine, University of Copenhagen, Copenhagen, DK 2100, Denmark; Faculty of Health Sciences, Forensic Medicine, University of Copenhagen, Copenhagen, DK 2100, Denmark; Faculty of Health Sciences, Forensic Medicine, University of Copenhagen, Copenhagen, DK 2100, Denmark

## Abstract

Treatment with antipsychotics such as chlorprothixene, clozapine, olanzapine, and quetiapine can be associated with cardiovascular and metabolic risks and poisoning, making them highly relevant in toxicological investigations. Since antipsychotics primarily act within the central nervous system, brain tissue serves as an alternative or complementary matrix to femoral blood in toxicological analysis. This study aimed to retrospectively evaluate brain concentrations and brain-blood ratios across toxic and non-toxic levels in 466 postmortem cases involving chlorprothixene, clozapine, olanzapine, and/or quetiapine. Median concentrations in brain tissue across all cases were 0.20 mg/kg for chlorprothixene, 4.2 mg/kg for clozapine, 0.42 mg/kg for olanzapine, and 0.34 mg/kg for quetiapine. Corresponding brain-blood ratios remained stable across the examined concentration range with median ratios of 3.1 for chlorprothixene, 2.9 for clozapine, 2.6 for olanzapine, and 3.0 for quetiapine. The provided brain concentrations and ratios can support further toxicological evaluations of postmortem cases.

## Introduction

Antipsychotic medications have transformed the management of severe mental disorders over the past seven decades. Their development began in 1954, when Elkes and Elkes published the first clinical study showing the effect of chlorpromazine on the behavior of psychotic patients [1]. Today, antipsychotics are widely used to manage conditions such as schizophrenia, bipolar mania and depression, autism spectrum disorders, and Tourette’s syndrome [2]. The expanded regulatory approval for indications outside psychosis and the increase of off-label use are linked with a global increase in antipsychotic prescriptions [3]. Among the antipsychotics most frequently prescribed and most often linked to intoxication are chlorprothixene, clozapine, olanzapine and quetiapine [4–10].

**Figure 1:**
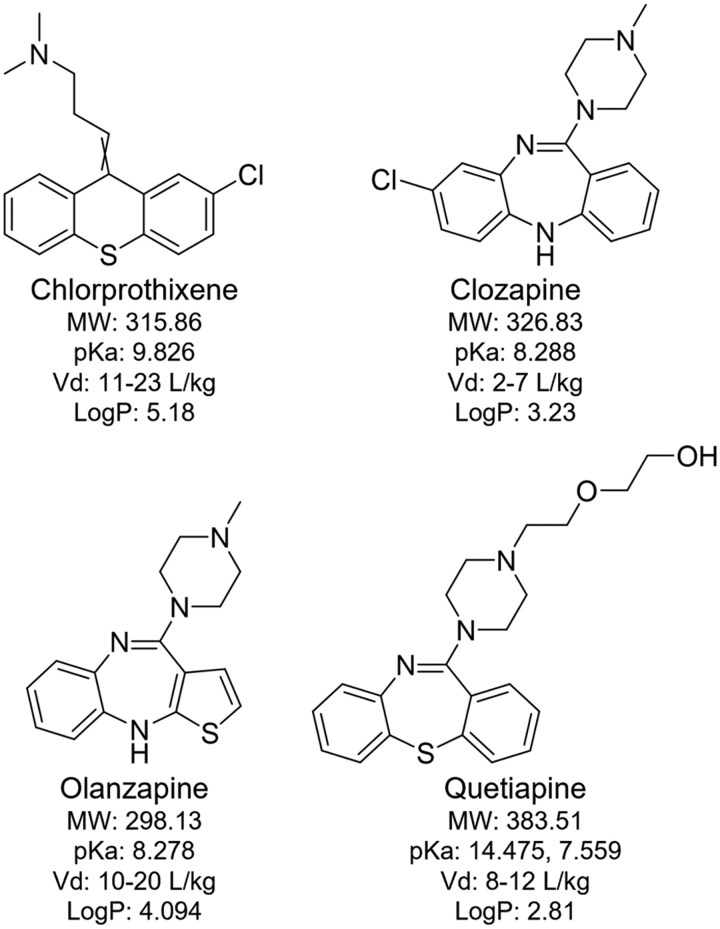
Chemical structures and selected properties of the four antipsychotics. Molecular weight and predicted pKa were calculated by ChemDraw (version 25.5.0), volume of distribution (Vd) was obtained from Baselt [11] and experimental LogP was obtained from PubChem.

Clozapine, olanzapine, and quetiapine are atypical antipsychotics with similar chemical structures (Fig. 1). Chlorprothixene, although a typical antipsychotic, has a pharmacological profile that is very similar to clozapine and olanzapine [10]. Those four antipsychotics are broad-spectrum, multiple receptor antagonist, targeting serotonin, and dopamine receptors, while also interacting with histaminergic, muscarinic, and adrenergic receptors [12, 13]. Due to their antagonism with the muscarinic M1 receptor, the antipsychotics can cause anticholinergic toxicity symptoms, including agitated delirium [14]. The high affinity for the serotonin 5-HT2A receptor associates the antipsychotics with high mortality of ischemic heart disease. Further, chlorprothixene, clozapine and quetiapine have a tendency for QT prolongation or torsades de pointes [5]. Hence, those antipsychotics are associated with high cardiovascular and metabolic risks, which can occur both at therapeutic doses and overdoses [10, 15]. For these reasons, and their association with unexplained and sudden deaths, antipsychotic drugs are highly relevant to investigate in forensic toxicology [16].

Traditionally, the contribution of antipsychotics to the cause of death is assessed by quantifying their concentration in peripheral whole blood, as this matrix is accessible and supported by reference concentrations in the literature [17, 18]. However, in some cases of advanced decomposition or severe blood loss, blood may be unavailable or unsuitable for analysis. Consequently, alternative matrices like brain, muscle, and liver tissue may be essential for toxicological analysis [19]. Among these matrices, brain tissue is especially promising due to its protected location and the high tendency of antipsychotics to accumulate in the brain, where high concentrations are reached even after short-term treatment [12, 16, 20]. However, the interpretation of concentrations in brain tissue is limited by a lack of reference concentration intervals that differentiate therapeutic from toxic levels. Therefore, this study aims to retrospectively investigate postmortem cases involving chlorprothixene, clozapine, olanzapine, and/or quetiapine, to evaluate brain concentrations and brain–blood ratios, supporting the use of brain tissue as an alternative or complementary matrix in toxicological analysis.

## Materials and methods

### Autopsy samples

Upon arrival at the Department of Forensic Medicine in Copen­hagen, human remains were stored at 5°C until autopsy, which was usually performed within four days. During postmortem examination, one or two tubes of 4–8 ml of femoral blood were collected without ligation of the iliac vein and preserved with 100 mg sodium fluoride and 22.5 mg potassium oxalate. Brain tissue was sampled from the cerebral cortex of the frontal lobe, ranging from 5 to 20 g. All biological material was stored at −20°C prior to standard toxicological analysis [21, 22].

### Toxicological analysis

Before toxicological analysis, 500 mg of grey matter was manually separated from the brain sample and homogenized in water (1:3). Sample preparation of brain homogenate and femoral blood included a fully automated polymeric strong cation solid-phase extraction (Phenomenex, Denmark) performed on a Tecan Freedom EVO 200 robotic platform (Tecan, Switzerland) or a Hamilton Vantage 2.0 liquid handling system (Hamilton, Switzerland). The antipsychotics were quantified with a validated method using ultra-high performance liquid chromatography–tandem mass spectrometry (UHPLC–MS/MS) as previously published by Nielsen *et al.* [23]. Calibrators in blood and quality control samples in both blood and brain tissue, along with deuterated internal standards, were used to assure accurate quantification. Key validation parameters have been reported in previous studies [23, 24]. In the present study, method performance parameters, including measurement range, accuracy, and imprecision (CV), were assessed through proficiency tests in whole blood and serum, as well as QC samples in whole blood and brain tissue (Table 1).

**Table 1:** Summary of method performance parameters for the four antipsychotics.

		Proficiency test		QC low blood (0.01 mg/kg)		QC high blood (0.5 mg/kg)		QC brain (0.25 mg/kg)	
	Measurement range (mg/kg)	*N*	*Z*-value^a^	*N*	CV (%)	*N*	CV (%)	*N*	CV (%)
Chlorprothixene	0.005–1.0	2	−0.3, −0.1	58	9.4	78	7.1	85	10.6
Clozapine	0.005–0.20	2	−0.8, −0.5	58	9.3	78	8.0	89	11.6
Olanzapine	0.010–1.0	2	−0.3, −0.3	58	10.6	78	7.8	89	8.4
Quetiapine	0.005–0.20	4	−0.6, −0.1, −0.1, −0.1	58	10.7	79	7.5	91	10.2

a A *Z*-value within the range of ±2 meets the acceptance criteria.

### Case inclusion criteria and categorization

During the medicolegal examination, the potential role of the antipsychotic to the cause of death was evaluated by two forensic toxicologists based on the analytical findings. The final cause of death was determined by the forensic pathologist, who took case circumstances into account. For this study, these conclusions and toxicological details from autopsy cases investigated between late 2014 and early 2026 were extracted from the laboratory information management system (LIMS). Cases were only included if they were quantified in both blood and brain tissue for at least one of the selected antipsychotics; chlorprothixene, clozapine, olanzapine, and/or quetiapine. Subsequently, positive results were grouped by antipsychotic, and a forensic toxicologists assigned each case to Class A, B, or C based on the determined cause of death and remarks on toxicity. Class A included cases in which the antipsychotic was the sole cause of death. In class B, the antipsychotic was contributing to the cause of death in combination with other pharmaceuticals, drugs of abuse, alcohol, or disease, and in class C the antipsychotic did not contribute to the cause of death.

### Statistical analysis

The median and 10th–90th percentile intervals were calculated for each antipsychotic within the A, B, and C classification, as well as within all cases and across the sum of class A and B. Differences in concentrations and brain–blood ratios between classes A, B, and C were calculated using the Wilcoxon rank-sum test or the Kruskal-Wallis test when calculating differences across more than two groups. The statistical program R, version 4.4.3, was used to determine statistical parameters.

## Results and discussion

In total, 557 paired blood and brain concentration datasets from 466 autopsy cases were included in the evaluation of brain concentrations and brain–blood ratios: quetiapine was detected in 226 cases, olanzapine was detected in 160 cases, chlorprothixene was detected in 83 cases, and clozapine was detected in 44 cases. Median and 10th–90th percentiles for the blood concentrations, brain concentrations, and brain–blood ratios are presented in Table 2. Due to the limiting and differing number of cases in the respective classes, the cases were grouped into class A + B, where the antipsychotic was the sole cause of death (class A) or where the antipsychotic was contributing to the cause of death in combination with other drugs or disease (class B), and class C, where the antipsychotic was not involved in the cause of death (Table 2).

**Table 2:** Femoral blood and brain tissue concentrations (mg/kg) and brain–blood ratios in postmortem cases across toxic (class A and B) and non-toxic (class C) cases for the selected antipsychotics.

			Blood concentration (mg/kg)		Brain concentration (mg/kg)		Brain–blood ratio	
Antipsychotic	Class	*N*	Median	10th–90th percentiles	Median	10th–90th percentiles	Median	10th–90th percentiles
Quetiapine	A + B	33	0.74	0.090–13	2.9	0.38–26	3.3	1.2–4.6
	C	193	0.088	0.013–0.77	0.26	0.033–2.4	2.9	1.6–5.0
Olanzapine	A + B	36	0.39	0.11–1.0	0.70	0.24–2.6	2.0	1.0–4.0
	C	124	0.12	0.039–0.36	0.32	0.063–0.86	2.7	1.2–4.7
Chlorprothixene	A + B	12	0.18	0.062–1.3	0.75	0.14–5.5	3.2	1.8–11
	C	71	0.056	0.007–0.16	0.17	0.020–0.88	3.1	1.5–7.2
Clozapine	A + B	20	3.1	1.4–7.1	8.5	3.4–17	3.0	1.5–3.7
	C	24	1.3	0.16–2.8	2.9	0.59–8.2	2.8	1.4–5.0

### Quetiapine

Quetiapine was quantified in both blood and brain tissue in 226 postmortem cases during the ten-year study period. Across all cases, the median blood concentration was 0.10 mg/kg (10th–90th percentiles: 0.015–1.5 mg/kg), which is consistent with values reported in earlier studies [25–29], as shown in Table 3. However, this median is lower than the median at 0.51 mg/L reported by Ketola *et al.* based on a larger dataset of 1792 cases [30]. The difference may be explained by the higher analytical lower limit of quantification (LLOQ) applied in the study by Ketola *et al.* (0.2 mg/L) compared to the applied LLOQ in the present study (0.005 mg/kg).

**Table 3:** Reported literature values (medians and 10th–90th percentiles) of the investigated antipsychotics in peripheral blood and brain tissue (mg/kg).

		Blood concentration (mg/kg)			Brain concentration (mg/kg)			Brain–blood ratio			
Antipsychotic	Class	*N*	Median	10th–90th percentiles	*N*	Median	10th–90th percentiles	*N*	Median	10th–90th percentiles	Refs.
Quetiapine	Unknown^a^	1792	0.51	0.2–6.4^b^							[30]
		100	0.16	0.02–1.38							[25]
		52	0.11	0.028–15^c^							[26]
		30	0.08	0.011–1.7^c^							[27]
		20	2.05	0.26–10.4							[31]
		17	6.0	0.44–16.2							[32]
		17	0.43								[33]
		16	0.06	0.015–1.14							[29]
		13	0.15	0.013–9.54	12	0.62	0.038–46.1	11	2.6	1.6–8.8	[28]
		10	0.24	0.037–19.3^c^							[34]
		10	1.05	0.027–9.2							[35]
		7	0.31	0.01–0.67	11	0.21	0.05–2.4	6	2.03	0.35–3.3	[36]
		6	2.0	0.26–11.25	2	20.7	4.53–36.9	1	2.07		[37]
		5	0.26	0.1–1.22							[38]
		3	0.80	0.070–0.88^c^							[39]
		1	0.048								[40]
					7	0.03	0.02–9.8				[16]
	A	9	8.1	0.7–22.0							[41]
		1	1.9								[26]
	B	18	3.85	0.97–41.6							[41]
		1	0.25								[26]
	C	32	0.079	0.011–0.27							[25]
		16	0.15	0.1–0.65							[41]
		10	0.04	0.0049–0.17							[26]
		8	0.29	0.20–0.60							[31]
	A + B	223	8.2	2.4–42							[42]
		12	3.5	2.01–13.6							[31]
		9	2.38	1.08–5.69							[43]
Olanzapine	Unknown^a^	2215	0.20	0.05–0.80^b^							[30]
		58	0.13	0.01–5.2^c^							[44]
		53	0.053	0.002–2.6^c^							[27]
		31	0.13	0.043–0.31							[25]
		28	0.44	0.14–3.02							[45]
		25	0.16	0.016–1.32^c^							[34]
		15	0.37								[33]
		14	0.24	0.14–0.73							[46]
		2	0.033	0.032–0.034							[39]
					3	2.01	0.90–27.1				[16]
	A	10	0.55	0.29–2.71							[41]
		6	3.3	1.17–5.2							[45]
	B	37	0.4	0.2–0.9							[41]
		12	0.7	0.14–0.97							[45]
		1	1.1		1	2.1		1	1.9		[47]
	C	94	0.1	0.06–0.2							[41]
		11	0.06	0.039–0.25							[25]
		10	0.22	0.13–0.41							[45]
	A + B	148	1.7	0.46–6.2							[42]
		9	0.89	0.77–1.54							[43]
Chlorprothixene	Unknown^a^	755	0.3	0.1–2.6^b^							[30]
		32	0.076	0.0072–1.0^c^							[26]
		1	0.0092								[40]
	A	5	1.9	1.6–3.3							[41]
	B	10	1.1	0.69–3.13							[41]
		1	0.164								[48]
	A + B	117	2.9	0.98–11							[42]
Clozapine	Unknown^a^	880	1.1	0.1–4.3^a^							[30]
		10	1.3	0.62–3.9							[25]
					4	4.79	1.31–6.76				[16]
	A	35	2.4	1.24–18.0							[41]
		7	8.2	3.7–12							[49]
		1	1.13								[25]
	B	22	2.65	1.4–22.0							[41]
	C	43	1.9	0–7.7							[49]
		26	0.4	0.09–1.55							[41]
		1	0.46								[25]
	A + B	120	5.2	1.9–20							[42]
		3	1.08	1.06–1.62							[43]

a Unknown included cases where the original sources did not report, specify, or allow classification, including instances of aggregated (all-cause) reporting.

b Limit of quantification—90th percentile.

c Concentration range.

The median concentration in brain tissue was approximately three times higher, at 0.34 mg/kg (10th–90th percentiles: 0.037–4.1 mg/kg), giving median brain–blood ratios of 3.0 (10th–90th percentiles: 1.5–5.0). Although Breivik *et al.* reported higher brain concentrations across 11 cases (range: 0.018–47.4 mg/L), brain–blood ratios (range: 1.6–11) were comparable with our study [28]. In contrast, Vignali *et al.* reported lower brain concentrations (range: 0.0378–2.4 mg/L) across 10 cases, and lower brain–blood ratios (range: 0.2–3.5) across 6 cases compared to our results [36].

In this study, most cases (*n* = 193) involved concentrations in which quetiapine did not contribute to the cause of death (class C), while in 33 cases quetiapine was considered a contributing ­factor (Table 3). In those 33 cases, quetiapine blood concentrations were lower than those reported in a larger study by Kriikku and Ojanperä, which included 223 cases involving both poly-drug and single drug poisonings with 10th–90th percentiles ranging from 2.4 to 42 mg/kg [42]. The higher concentrations in that study likely reflect its exclusion of cases in which quetiapine contributed to the cause of death but was not registered as principal finding, whereas our study included such cases in class B. Quetiapine was the sole cause of death (class A) in five cases, where 10th–90th percentiles in blood and brain ranged from 4.86 to 500 mg/kg (median: 15 mg/kg) and 13–108 mg/kg (median: 44 mg/kg), respectively. In the remaining 28 cases quetiapine contributed to the cause of death in combination with other drugs, alcohol or diseases (class B), giving a median concentration of 0.48 mg/kg (10th–90th percentiles: 0.077–4.9 mg/kg) in blood and 2.0 mg/kg (10th–90th percentiles: 0.33–12 mg/kg) in brain tissue. Among the cases in class B, quetiapine was most frequently found in combination with the opioid methadone, followed by the benzodiazepines nordazepam and oxazepam. Quetiapine and methadone are frequently co-administered, reflecting high prevalence of substance misuse among individuals with certain mental illnesses [50]. Further, Uehlinger *et al.* showed that quetiapine may increase methadone’s plasma concentrations [51], but evidence for additive effects was not found [52].

### Olanzapine

Olanzapine was quantified in both blood and brain in 160 cases, of which 40 were reported in a previous study by Nedahl *et al.* [24]. The median blood concentration across the cases was 0.16 mg/kg (10th–90th percentiles: 0.040–0.58 mg/kg), which is consistent with the median concentrations reported in earlier studies (Table 3) [34, 44]. In brain tissue, the 10th–90th percentiles ranged from 0.080 to 1.1 mg/kg with a median at 0.42 mg/kg, resulting in a median brain-blood ratio of 2.6 (10th–90th percentiles: 1.1–4.6).

Of the 160 cases, 36 cases involved concentrations of olanzapine, where olanzapine contributed to the cause of death, while 124 cases involving olanzapine were categorized as class C. Across classes A and B, the median blood concentration (Table 2) was lower than median concentrations found in other studies due to other inclusion criteria [42, 43]. In eight cases, olanzapine was the sole cause of death (class A), with blood concentrations of 0.50–2.3 mg/kg (median: 1.0 mg/kg) and brain concentrations of 1.0–4.1 mg/kg (median: 2.2 mg/kg). In the remaining 28 cases, the cause of death involved olanzapine in combination with other substances or health conditions (class B), with median concentrations in blood and brain tissue of 0.32 and 0.55 mg/kg, respectively. The polydrug poisonings primarily involved olanzapine with methadone, clonazepam, or ethanol. The frequent co-occurrence of methadone and clonazepam likely reflects methadone’s prominence in toxicological cases and the heightened risk of depression in the central nervous and respiratory system when olanzapine is combined with clonazepam [53, 54].

### Chlorprothixene

In 83 cases, chlorprothixene was quantified in both blood and brain tissue. The median concentration in blood across those cases was 0.072 mg/kg (10th–90th percentiles: 0.007–0.29 mg/kg), which aligns with concentrations found in an earlier study by Mikkelsen *et al.* [26], but is lower than those reported in a larger study by Ketola *et al.* [30], likely reflecting the higher LLOQ applied in that study. In brain tissue, the median concentration across all cases was 0.20 mg/kg (10th–90th percentiles: 0.021–0.10 mg/kg), giving a median brain–blood ratio of 3.1 and 10th–90th percentiles ranging from 1.5 to 7.4). In 71 of the cases, chlorprothixene was not involved in the cause of death (class C), while the remaining 12 cases involving chlorprothixene were within class A and B (Table 2). However, due to a limited number of cases, detailed data are not presented here.

### Clozapine

Clozapine was quantified in both blood and brain tissue in 44 cases; in 20 cases clozapine was considered a contributing factor, and in 24 cases clozapine was not involved in the cause of death. Blood concentrations across all cases ranged from 0.28 to 4.9 mg/kg with a median at 1.7 mg/kg, aligning with concentration ranges found in earlier studies [25, 30]. Concentrations in brain tissue were considerably higher, with a median of 4.2 mg/kg (10th–90th percentiles: 1.2–14 mg/kg) and a median brain–blood ratio of 2.9 (10th–90th percentiles: 1.4–4.0). Our concentrations in brain tissue correspond with four cases previously reported by Sampedro *et al.* where the median was 4.8 mg/kg and the 10th–90th percentiles ranged from 1.3 to 6.8 mg/kg [16].

In 12 cases, clozapine was found as the sole cause of death (class A), while in the remaining 8 cases, clozapine contributed to the cause of death in combination with other drugs or diseases (class B). Across the cases in class A, the median concentrations in blood and brain tissue were 4.2 mg/kg (10th–90th percentiles: 2.1–9.1 mg/kg) and 9.8 mg/kg (10th–90th percentiles: 3.5–17 mg/kg), respectively. Median brain–blood ratios were 2.1 with 10th–90th percentiles ranging from 1.4 to 3.1. Among class B cases, the median blood concentration was 1.7 mg/kg (10th–90th percentiles: 1.1–3.3 mg/kg), while the median was 7.3 mg/kg in brain tissue (10th–90th percentiles: 3.5–11 mg/kg). In those cases, clozapine was found primarily together with methadone or other antipsychotics.

In the 44 cases, metabolite-parent ratios of norclozapine and clozapine were calculated, resulting in a median metabolite–parent ratio of 0.49 (10th–90th percentiles: 0.27–0.90) in blood and of 0.70 (10th–90th percentiles: 0.29–1.4) in brain tissue. The lower metabolite–parent ratio in blood, could possibly be explained by the difference in chemical composition between clozapine and its metabolite or clozapine’s greater tendency for postmortem redistribution compared to norclozapine [49], which would increase the concentration of clozapine in blood more than the concentration of norclozapine.

### Toxicity patterns

Among the included antipsychotics, clozapine showed the highest proportion of cases (45%) where the antipsychotic contributed to the cause of death. Olanzapine contributed to the cause of death in about a quarter of the cases, while quetiapine and chlorprothixene were involved in the cause of death in about 15% of the cases. The high proportion for clozapine likely reflects its narrow therapeutic index ranging from 0.35 to 0.6 mg/L [55], its higher toxicity compared to quetiapine and olanzapine [56] and its use in treatment-resistant schizophrenia and other severe mental illnesses [8, 56].

**Figure 2:**
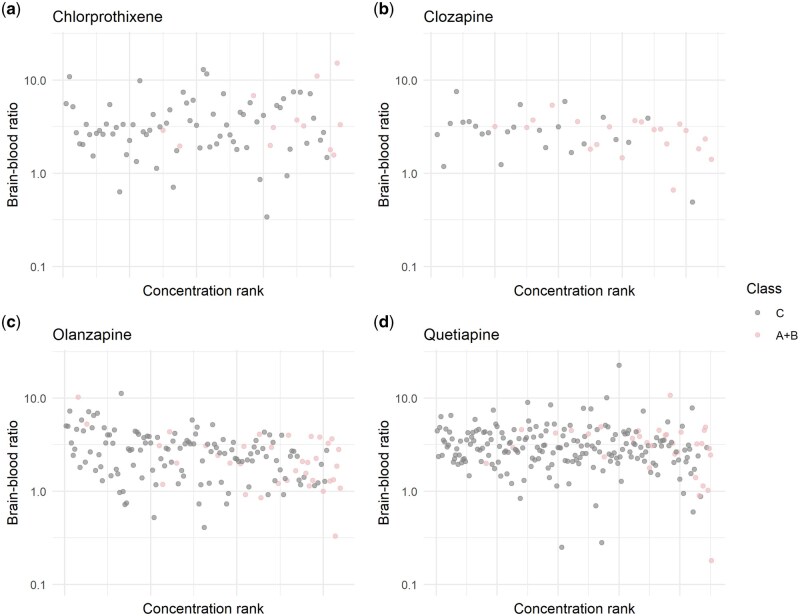
Relationship between the concentrations in blood of the selected antipsychotics and their brain–blood ratios. (a) Chlorprothixene, (b) clozapine, (c) olanzapine, and (d) quetiapine. The graph shows the ranked concentrations in blood (linear scale) against the corresponding brain–blood ratios (logarithmic scale).

As expected, based on the classification criteria, the cases in which the antipsychotics were contributing to the cause of death, had higher concentrations in blood than the cases in which the antipsychotic was not involved in the cause of death. However, as seen in Fig. 2, there was an overlap between concentrations in class C compared to cases in class A and B. High concentrations in class C could be explained by other evident causes of death, such as overdose by another substance or underlying disease. Furthermore, in class B, therapeutic concentrations may have contributed to the fatal outcome due to potential poly-drug interactions and additive pharmacological effects, which may explain the overlap between concentrations in class B and class C.

In poly-drug intoxication cases (class B), the most frequently co-detected substances were methadone, benzodiazepines, and ethanol. This aligns with previous findings that benzodiazepines and ethanol are commonly encountered in individuals with severe mental disorders [4, 57]. This is plausible due to clinical practice, the combination of antipsychotics and benzodiazepines in agitation in emergency departments, or the potential of additive effects of sedative medications (opioids, benzodiazepines, or alcohol) with atypical antipsychotics [58–60]. Since many antipsychotics are central nervous system depressants, toxicity risk increases when taken in combination with alcohol and/or opioids [61, 62].

### Brain–blood ratios

Wilcoxon rank tests showed no significant differences in the brain–blood ratios between classes A, B, and C for any antipsychotic. As the ratios remain independent of class (Fig. 2), the observed brain-blood ratios demonstrate that brain tissue is a reliable matrix for interpretation when blood is unavailable or unsuitable for analysis. However, brain–blood ratios tend to be slightly lower in cases where the antipsychotic was considered the sole cause of death. Interestingly, brain–blood ratios did not significantly differ between the antipsychotics, except for olanzapine which had a lower brain–blood ratio compared to chlorprothixene (*P* < .01) and quetiapine (*P* < .001). The lower ratio for olanzapine could potentially be explained by olanzapine’s high susceptibility to postmortem redistribution or its instability compared to the other antipsychotics, giving higher concentrations in blood compared to brain tissue [63, 64]. No significant difference was found between olanzapine and clozapine, which could be due to their similar chemical structure and receptor-binding profiles [65].

### Challenges and limitations

Toxicological measurement in brain tissue is challenged by the complex, heterogeneous matrix and variable lipid composition across tissue types (e.g. white and grey matter). In cases of advanced postmortem degradation, separation between these tissues was not possible. In such cases, mixed samples were analyzed, which may have influenced analyte concentrations due to differences in matrix effects and extraction efficiencies, despite the use of isotopically labeled internal standards. The use of blood-based calibrators for the quantification of antipsychotics may have introduced some uncertainty, although previous work has demonstrated its suitability and reliability for this approach [23].

Additionally, the relatively low number of A cases limited conclusions regarding sole intoxications, and the lack of information regarding postmortem interval prevented assessment of postmortem redistribution effects. The interpretation may have been further complicated by potential additive or interactive effects between different drugs, thereby introducing uncertainty in case classification. Finally, direct comparison between studies was limited by differences in sampling strategies, analytical methodologies, and classification criteria, including insufficient reporting of tissue separation and anatomical origin.

Despite these challenges and limitations, brain–blood ratios remain useful and can aid in the interpretation of toxicological findings in postmortem cases, and this study contributes to the existing literature by providing new data.

## Conclusion

When blood samples are unavailable, concentrations in brain tissue can serve as reference values. This study provided postmortem concentrations for four commonly prescribed antipsychotics in both brain tissue and femoral blood across non-toxic and toxic levels from 466 cases. The concentrations in blood and brain tissue showed a good correlation with brain–blood ratios of approximately 3 across the investigated concentration range for all antipsychotics. The individual median brain–blood ratios were 3.1 for chlorprothixene, 2.9 for clozapine, 2.6 for olanzapine, and 3.0 for quetiapine. These established concentrations in brain tissue and brain-blood ratios will support future forensic investigations when using brain tissue as an alternative or complementary matrix.

## Data Availability

The data underlying this article cannot be shared publicly due to the nature of the study, which involves retrospective analysis of postmortem material collected from routine cases. Data sharing is limited to protect confidentiality and is per institutional guidelines.
